# Esketamine for negative emotions and cognitive function in general anesthesia: a systematic review and meta-analysis

**DOI:** 10.3389/fmed.2026.1816437

**Published:** 2026-06-12

**Authors:** Xiaofan Yang, Jing Qin, Wenqing Xu

**Affiliations:** Department of Anesthesiology, Tinglin Hospital, Shanghai, China

**Keywords:** esketamine, general anesthesia, meta-analysis, postoperative neuropsychiatric complications, systematic review

## Abstract

**Introduction:**

This study aims to systematically evaluate, through a systematic review and meta-analysis, the effect of esketamine on postoperative negative emotions (anxiety and depression) and cognitive function in patients undergoing general anesthesia.

**Methods:**

Following PRISMA guidelines, we systematically searched PubMed, Web of Science, Embase, Cochrane Library, and CNKI to identify studies up to April 2025. Included randomized controlled trials (RCTs) involved adults undergoing general anesthesia, comparing perioperative esketamine to placebo/standard care, and reporting outcomes on negative emotions or cognitive function. Risk of bias was evaluated using Cochrane RoB 2.0, and Data were synthesized using R 4.3.3 with random-effects models; heterogeneity and robustness were assessed via I^2^ statistics and sensitivity analyses.

**Results:**

Thirteen RCTs involving 1,312 patients were included. Esketamine significantly reduced depressive (SMD = −0.92, 95% CI: [−1.39, −0.46]) and anxiety symptoms (SMD = −1.23, 95% CI: [−1.71, −0.75]), with low certainty of evidence for both outcomes. Cognitive outcomes were heterogeneous: MMSE showed no significant effect overall (SMD = 0.22, 95% CI: [−0.87, 1.30]) but became significant after excluding an outlier (SMD = 0.65, 95% CI: [0.39, 0.90]), while MoCA results remained non-significant (SMD = 0.93, 95% CI: [−0.99, 2.84]), and the certainty of evidence for MMSE and MoCA was rated very low.

**Conclusion:**

Esketamine demonstrates robust antidepressant and anxiolytic effects postoperatively, while its cognitive benefits remain unclear owing to high heterogeneity and very low evidence certainty. Although these findings support the potential application of esketamine in perioperative emotional management, the low to very low overall certainty of evidence justifies the need for further high-quality, large-scale RCTs to clarify its cognitive effects and establish optimal dosing regimens.

**Systematic review registration:**

Identifier, CRD420251078985.

## 1 Introduction

Postoperative neuropsychiatric complications, particularly negative emotional states such as anxiety and depression, as well as postoperative cognitive dysfunction (POCD), are increasingly recognized as critical determinants of surgical outcomes and long-term recovery (1, 2). With the global aging population and the rising volume of surgeries performed under general anesthesia, these complications have become prevalent across diverse surgical populations, especially among the elderly (3). POCD, a multifactorial and often insidious condition, is characterized by impairments in memory, attention, executive function, and processing speed (4). Its presence is associated with delayed recovery, reduced quality of life, prolonged hospitalization, increased risk of dependency, and even elevated mortality (2). Similarly, perioperative emotional disturbances can interfere with rehabilitation, pain control, and adherence to postoperative regimens, compounding patient morbidity (5).

Despite mounting awareness of these issues, current perioperative management strategies remain suboptimal. Traditional interventions such as anxiolytics or cognitive enhancers are limited by inconsistent efficacy and potential adverse effects. Against this backdrop, esketamine, the S-enantiomer of ketamine, has emerged as a novel candidate for perioperative neuroprotection (6). Esketamine exhibits higher affinity for the N-methyl-D-aspartate (NMDA) receptor than its racemic counterpart and offers potent analgesic, dissociative, and antidepressant effects at subanaesthetic doses (7). Notably, esketamine has received regulatory approval in several countries for the treatment of treatment-resistant depression, underscoring its robust and rapid antidepressant properties (6). Mechanistically, its neuroprotective potential is thought to involve modulation of glutamatergic transmission, reduction of neuroinflammation, and enhancement of synaptic plasticity through brain-derived neurotrophic factor signaling pathways (8).

Preliminary clinical studies have suggested that intraoperative or early postoperative administration of low-dose esketamine may ameliorate emotional distress and cognitive decline following surgery (9, 10). However, the evidence remains fragmented, with considerable heterogeneity in study designs, patient populations, dosing regimens, and outcome measures. Some trials report significant improvements in mood and cognition, whereas others observe minimal or transient effects (11, 12). Furthermore, questions persist regarding the optimal timing, route of administration, and safety profile of esketamine in the surgical setting. Therefore, we performed a systematic review and meta-analysis of randomized controlled trials to examine the impact of esketamine on postoperative negative emotions and cognitive function in patients undergoing general anesthesia. Our goal was to clarify its potential role as a perioperative neuropsychiatric modulator and to inform future clinical decision-making and research directions.

## 2 Methods

### 2.1 Study design and registration

This study was conducted in accordance with the Preferred Reporting Items for Systematic Reviews and Meta-Analyses (PRISMA) guidelines. The protocol was registered prospectively in the PROSPERO database (Registration ID: CRD420251078985), ensuring methodological transparency and reproducibility.

### 2.2 Search strategy

We systematically searched five major electronic databases including PubMed, Web of Science, Embase, the Cochrane Library, and CNKI for studies published from database inception to April 1st, 2025. The search focused on the use of esketamine or S-ketamine in patients undergoing general anesthesia, with outcomes related to negative emotions such as anxiety and depression and postoperative cognitive function. Only studies published in English or Chinese were included. Detailed search strategies for each database are presented in Appendix Table 1.

### 2.3 Eligibility criteria

We included RCTs that met the following criteria: (1) Adult patients (≥18 years old) undergoing surgery under general anesthesia. (2) Perioperatively administered esketamine compared to placebo or standard care; (3) At least one of the following measured postoperatively: negative emotional states (anxiety and/or depression) and/or cognitive function (using validated scales such as MMSE, MoCA).

### 2.4 Exclusion criteria included

(1) Non-randomized studies, reviews, conference abstracts, editorials, or case reports. (2) Animal studies or *in vitro* research. (3) Studies without full-text access or lacking relevant outcome data.

### 2.5 Data extraction

Two reviewers independently screened titles, abstracts, and full texts to identify eligible studies. Disagreements were resolved by discussion or consultation with a third reviewer. Data were extracted using a predesigned form, including study characteristics, participant demographics, surgical type, esketamine dosage and administration details and results.

Missing standard deviations were calculated from reported confidence intervals or *p*-values when available; studies with non-derivable missing outcome data were excluded from the relevant meta-analysis.

### 2.6 Risk of bias assessment

The methodological quality of all included randomized controlled trials was assessed independently by two reviewers using the Cochrane Risk of Bias 2.0 tool. The following five domains were evaluated: (1) bias arising from the randomization process, (2) bias due to deviations from intended interventions, (3) bias due to missing outcome data, (4) bias in measurement of the outcome, and (5) bias in selection of the reported result. Each domain was rated as “low risk,” “some concerns,” or “high risk” of bias. Disagreements between reviewers were resolved through discussion or consultation with a third reviewer. The overall risk of bias for each study was summarized and visualized using RevMan software. Studies rated as having high risk in multiple domains were noted and considered in sensitivity analyses.

### 2.7 Data synthesis and statistical analysis

Meta-analyses were performed using R (version 4.3.3). For continuous outcomes, standardized mean differences (SMD) with 95% confidence intervals (CIs) were calculated. Pooled estimates were then obtained using inverse-variance weighting to combine study-specific effect sizes. A random-effects model was applied due to anticipated clinical heterogeneity. Statistical heterogeneity was assessed using the I^2^ statistic, with thresholds of 25, 50, and 75% representing low, moderate, and high heterogeneity, respectively. Sensitivity analyses were conducted using leave-one-out approach, in which each study was sequentially removed to assess its individual influence on the pooled effect estimate. In addition to primary analyses, clinically relevant subgroup analyses were performed, to further explore potential sources of heterogeneity. Evidence for all outcomes was evaluated using Grading of Recommendations Assessment, Development and Evaluation (GRADE) and categorized into high, moderate, low, or very low certainty (13).

## 3 Results

### 3.1 Literature search results and general characteristics

**Figure 1:**
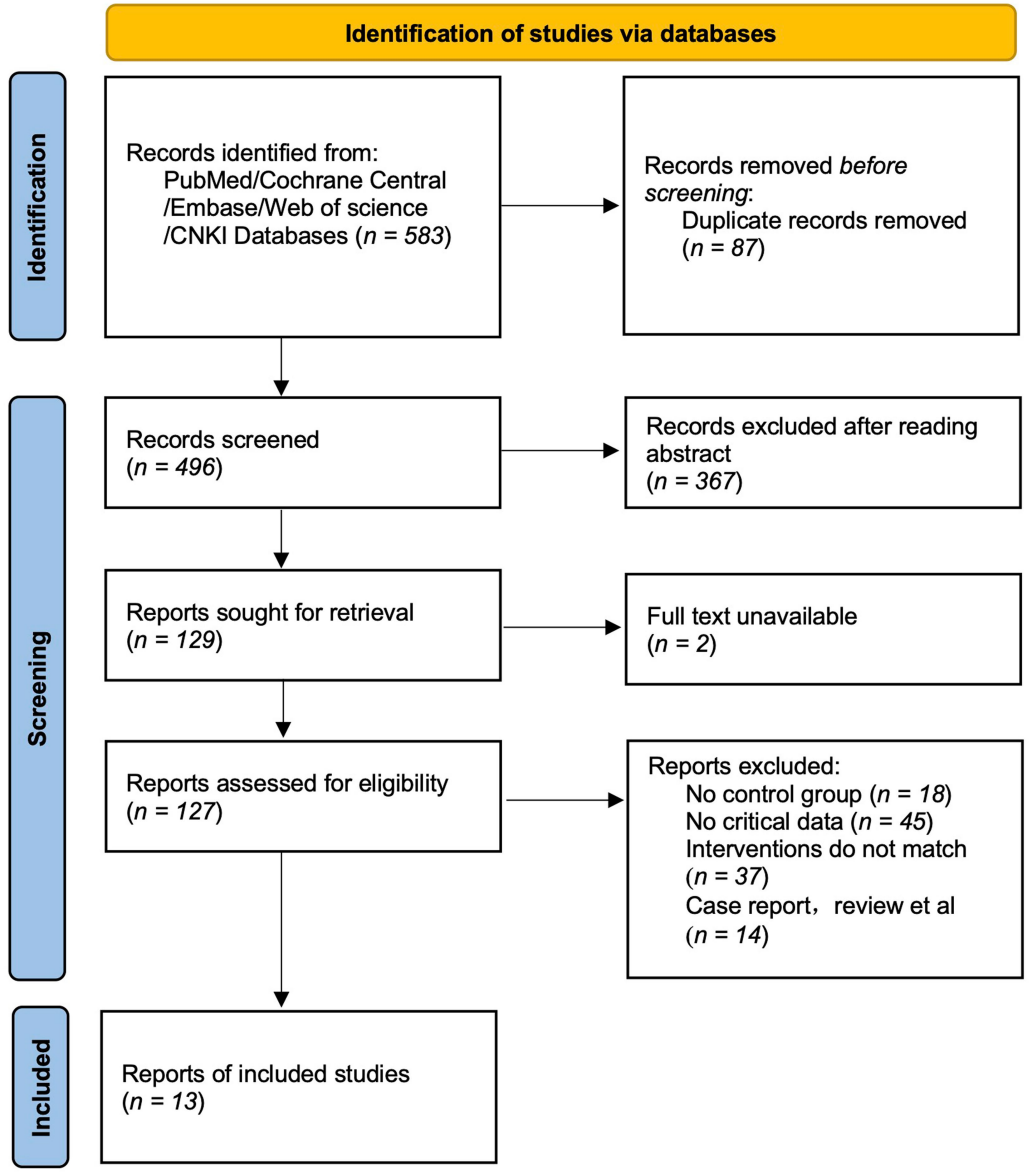
Flow chart.

A total of 583 studies were initially identified through database searches. After screening based on predefined inclusion and exclusion criteria, 13 RCTs (9, 10, 14–24) comprising a total of 1,312 patients were included in the final analysis. The study selection process is depicted in Figure 1, and the main characteristics of the included studies are summarized in Table 1.

**Table 1:** Baseline characteristics.

First author	Years	Participants number		Age		Gender (male/female)		Surgical approach
		Intervention	Control	Intervention	Control	Intervention	Control	
Wang Xueli (14)	2022	87	88	50.01 ± 3.95	49.41 ± 3.73	0/87	0/88	Laparoscopic total hysterectomy
Sun Yufang (15)	2023	45	45	67.97 ± 4.93	68.34 ± 4.30	19/26	20/25	Lower extremity surgery
Ma Li (9)	2025	28	28	53.16 ± 2.61	53.79 ± 2.44	15/13	16/12	Cranial hematoma removal and/or decom- pressive craniectomy
Su (10)	2024	60	60	52.07 ± 8.63	52.19 ± 8.76	36/24	37/23	Radical resection of colorectal cancer
Ma Genshan (16)	2022	40	40	68.5 ± 6.1	66.9 ± 5.5	18/22	20/20	Laparoscopic radical resection of colon cancer
Wei Meng (17)	2024	40	40	53.79 ± 3.52	53.76 ± 3.55	0/40	0/40	Laparoscopic total hysterectomy
Tang Xiaolei (18)	2024	60	60	70.84 ± 4.78	70.17 ± 4.93	25/35	36/34	Hip fracture surgery
Xu Shan-hu (19)	2024	40	40	70.93 ± 3.95	71.55 ± 3.39	23/17	21/19	Thoracoscopic lobectomy
Wang Lingtong (20)	2024	45	44	70.56 ± 5.36	71.32 ± 5.58	17/28	16/28	Hip replacement
Zhou Jing (21)	2022	25	25	69.1 ± 1.3	69.2 ± 1.4	25/0	25/0	Transurethral resection of prostate
Jin Liang (22)	2024	76	76	26.85 ± 3.14	26.62 ± 3.10	0/76	0/76	Laparoscopic ectopic pregnancy surgery
Wei Jingjing (23)	2023	50	50	30.34 ± 4.21	30.62 ± 4.13	0/50	0/50	Laparoscopic surgery for infertility
D. Zhou (24)	2023	60	60	48.30 ± 11.19	47.33 ± 12.12	0/60	0/60	Breast and thyroid surgery

### 3.2 Risk of bias assessment in included studies

**Figure 2:**
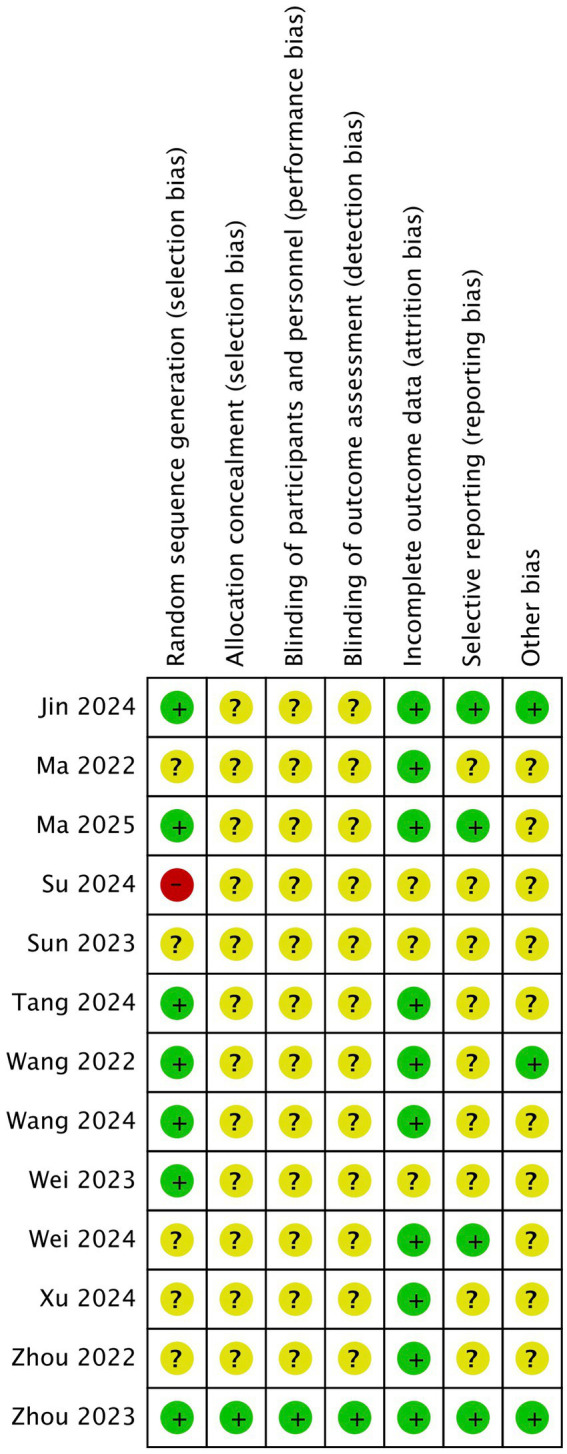
Risk of bias summary.

The risk of bias assessment of the included studies indicated that the majority exhibited unclear risk in critical domains of random sequence generation and allocation concealment. Regarding selective reporting, nine studies were rated as low risk, while the remaining four were classified as unclear risk. Overall, there was a degree of heterogeneity in methodological quality among the included studies, predominantly due to insufficient transparency in randomization procedures and blinding implementation (Figure 2).

### 3.3 Cognitive function assessment

**Figure 3:**
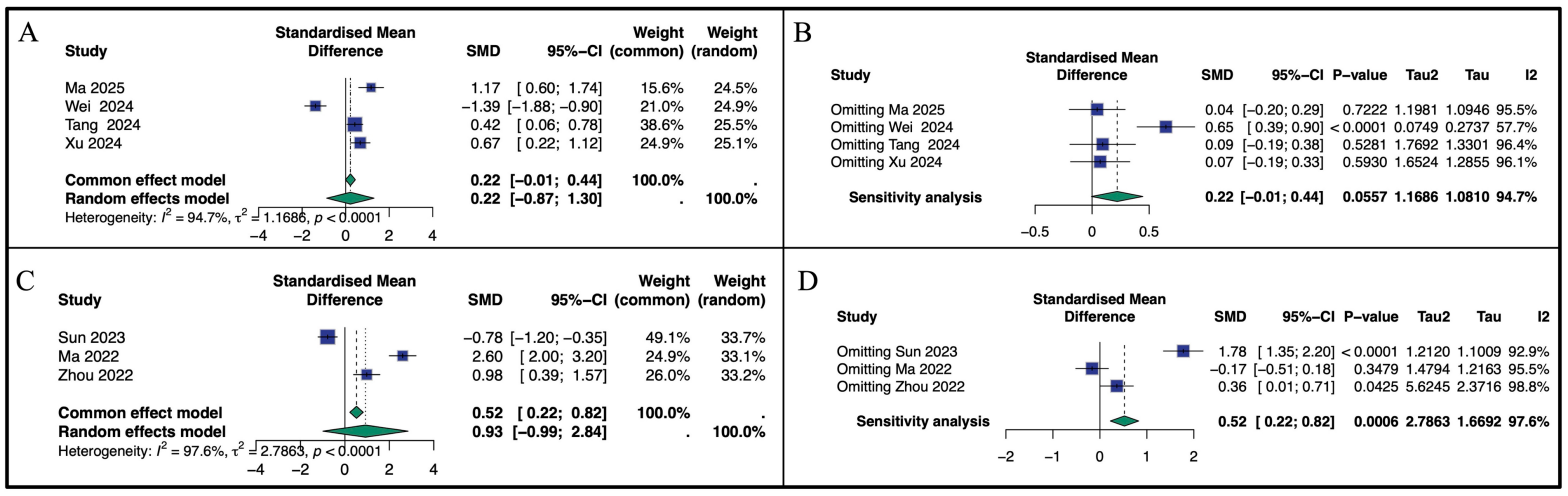
Cognitive function assessment. **(A)** MMSE meta; **(B)** Leave-one-out sensitivity analysis of MMSE; **(C)** MoCA meta; **(D)** Leave-one-out sensitivity analysis of MoCA. MMSE, Mini-Mental State Examination; MoCA, Montreal Cognitive Assessment; SMD, Standardized mean difference; CI, Confidence interval.

Cognitive function was assessed using MMSE and MoCA. Four studies including 336 patients employed the MMSE, and the random-effects meta-analysis showed a non-significant pooled effect size (SMD = 0.22, 95% CI: [−0.87, 1.30], *p* = 0.6967) with substantial heterogeneity (I^2^ = 94.7%). Wei et al. reported results that differed markedly from other studies, and after excluding this study, the combined effect became significant (SMD = 0.65, 95% CI: [0.39, 0.90], *p* < 0.0001), with reduced heterogeneity (I^2^ = 57.7%) (Figures 3A,B).

Similarly, three studies involving 220 patients assessed cognition using the MoCA, which also showed high heterogeneity (I^2^ = 97.6%). The random-effects model yielded a non-significant result (SMD = 0.93, 95% CI: [−0.99, 2.84], *p* = 0.3428) (Figures 3C,D).

### 3.4 Depressive symptoms assessment

**Figure 4:**
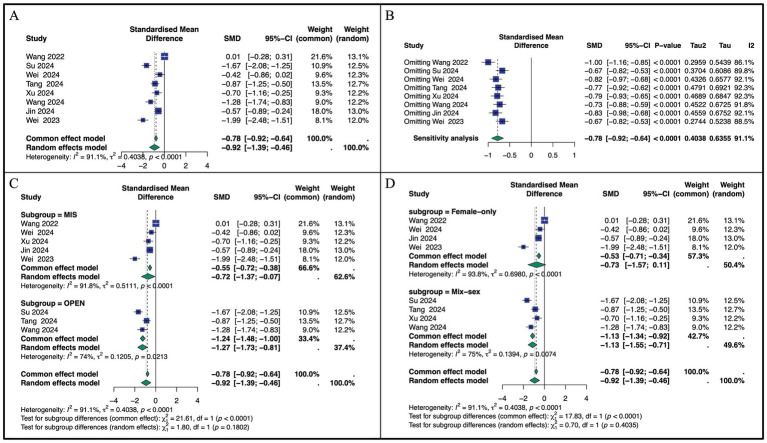
Depressive symptoms assessment. **(A)** SDS meta; **(B)** Leave-one-out sensitivity analysis of SDS; **(C)** Subgroup analysis by sugical type; **(D)** Subgroup analysis by sex. SDS, Self-Rating depression scale; SMD, Standardized mean difference; CI, Confidence interval.

Depressive symptoms were evaluated using the Self-Rating Depression Scale (SDS) across eight studies involving 926 patients. Meta-analysis demonstrated a significant reduction in SDS scores in patients receiving esketamine compared to controls (random-effects model: SMD = −0.92, 95% CI: [−1.39, −0.46], *p* < 0.0001). This effect size corresponds to a large magnitude of effect and suggests a substantial reduction in depressive symptoms, approximately equivalent to a decrease of about 5–10 points on the SDS scale based on typical standard deviations reported in perioperative populations. Sensitivity analysis confirmed the robustness of these findings, with pooled effect sizes ranging from −1.00 to −0.67 after removal of individual studies. Considerable heterogeneity was observed (I^2^ = 86.1–92.1%) (Figures 4A,B).

In subgroup analysis by surgical type, the effect size was −0.55 (95% CI: −0.72 to −0.38; I^2^ = 91.8%) for minimally invasive surgery and −1.24 (95% CI: −1.48 to −1.00; I^2^ = 74.0%) for open surgery, with a significant between-group difference (*p* < 0.0001) (Figure 4C). In sex-based subgroups, the pooled effect was −0.73 (95% CI: −1.57 to 0.11) in female-only studies and −1.13 (95% CI: −1.55 to −0.71) in mixed-sex studies, with a significant between-group difference (*p* < 0.0001) (Figure 4D).

### 3.5 Anxiety symptoms assessment

**Figure 5:**
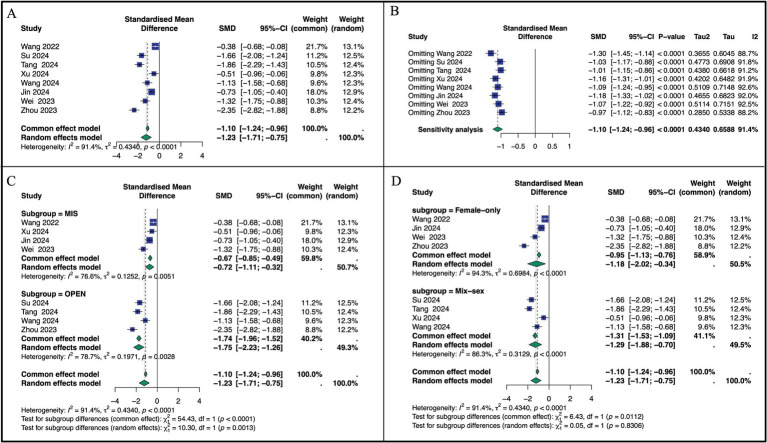
Anxiety symptoms assessment. **(A)** SAS meta; **(B)** Leave-one-out sensitivity analysis of SAS; **(C)** Subgroup analysis by sugical type; **(D)** Subgroup analysis by sex. SAS, Self-Rating Anxiety Scale; SMD, Standardized mean difference; CI, Confidence interval.

Anxiety symptoms were assessed via the Self-Rating Anxiety Scale (SAS) in eight studies including 966 patients. The meta-analysis showed significant improvements in anxiety following esketamine treatment (random-effects model: SMD = −1.23, 95% CI: [−1.71, −0.75], *p* < 0.0001). This represents a large effect size, indicating a marked reduction in anxiety symptoms, which may correspond to an approximate decrease of 6–12 points on the SAS scale when considering typical variability in clinical populations. Sensitivity analyses indicated stable results, with effect sizes varying between −1.30 and −0.97 after excluding individual studies. Heterogeneity remained high (I^2^ = 88.2–92.6%) (Figures 5A,B).

In subgroup analysis by surgical type, the effect size was −0.72 (95% CI: −1.11 to −0.32; I^2^ = 76.6%) for minimally invasive surgery and −1.75 (95% CI: −2.23 to −1.26; I^2^ = 78.7%) for open surgery, with a significant between-group difference (*p* = 0.0013) (Figure 5C). In sex-based subgroups, the pooled effect was −0.53 (95% CI: −0.71 to −0.34; I^2^ = 93.8%) in female-only studies and −1.13 (95% CI: −1.55 to −0.71; I^2^ = 75.0%) in mixed-sex studies, with a significant between-group difference (*p* < 0.0001) (Figure 5D).

### 3.6 GRADE rating

In this study, the certainty of evidence for the primary outcomes was evaluated using the GRADE approach (Table 2). The certainty of evidence for postoperative depressive and anxiety symptoms was rated as low, mainly downgraded due to unclear allocation concealment and blinding, inadequate methodological reporting, and substantial heterogeneity across included trials. The certainty of evidence for cognitive function (MMSE, MoCA) was rated as very low; in addition to the above limitations of bias and heterogeneity, serious imprecision caused by small sample sizes further downgraded the certainty. Although the meta-analysis suggests that esketamine has potential psychological and cognitive protective benefits, the findings should be interpreted with caution owing to the methodological quality and clinical heterogeneity of the available evidence.

**Table 2:** Evidence profile for each outcome.

No. of studies	Outcomes	Assessment	SMD	95% CI	Risk of bias	Inconsistency	Indirectness	Impression	Other consideration	Certainly
8	Depressive symptoms	SDS	−0.92	−1.39, −0.46	Serious^a^	Serious^b^	Not serious	Not serious	None	⨁⨁◯◯ Low^a,b^
8	Anxiety symptoms	SAS	−1.23	−1.71, −0.75	Serious^c^	Serious^b^	Not serious	Not serious	None	⨁⨁◯◯ Low^b,c^
4	Cognitive function	MMSE	0.22	−0.87, 1.3	Serious^a^	Serious^b^	Not serious	Serious^d^	None	⨁◯◯◯ Very low^a,b,d^
3	Cognitive function	MoCA	0.93	−0.99, 2.84	Serious^a^	Serious^b^	Not serious	Serious^d^	None	⨁◯◯◯ Very low^a,b,d^

CI, Confidence interval; SMD, Standardized mean difference; SDS, Self-Rating depression scale; SAS, Self-Rating anxiety scale; MMSE, Mini-Mental State Examination; MoCA, Montreal Cognitive Assessment.

^a^Unclear allocation concealment and blinding.

^b^Significant heterogeneity.

^c^Most studies had unclear methodological details.

^d^Small-sample size.

## 4 Discussion

This systematic review and meta-analysis included 13 randomized controlled trials involving 1,312 postoperative patients who underwent general anesthesia, aiming to evaluate the impact of esketamine on affective symptoms and cognitive performance in the perioperative period. Our findings suggest that esketamine has a significant and consistent effect in alleviating both depressive and anxiety symptoms, as assessed primarily by the SDS and SAS, respectively. However, its effects on cognitive function, measured via MMSE and MoCA, were less uniform and characterized by substantial heterogeneity across studies.

The antidepressant efficacy of esketamine observed in this analysis is consistent with accumulating evidence supporting its rapid-onset antidepressant properties in both psychiatric and perioperative populations. The neurobiological mechanisms underlying this effect are multifaceted. Esketamine acts as a noncompetitive antagonist of the NMDA receptor, which plays a central role in glutamatergic neurotransmission (25). Blockade of this receptor induces increased signaling through *α*-amino-3-hydroxy-5-methyl-4-isoxazolepropionic acid receptors, enhancement of brain-derived neurotrophic factor expression, and activation of the mammalian target of rapamycin pathway (26). Collectively, these downstream effects contribute to increased synaptic plasticity and neural connectivity in regions implicated in mood regulation, such as the prefrontal cortex and hippocampus (26). In the perioperative setting, where neuroinflammation, surgical stress, and general anesthesia may disrupt affective homeostasis, esketamine’s modulation of glutamatergic pathways may provide rapid and targeted symptom relief (6).

The beneficial effects on anxiety symptoms are similarly supported by neurophysiological evidence. Esketamine influences serotonergic and dopaminergic neurotransmission and exerts modulatory effects on limbic structures, including the amygdala and anterior cingulate cortex, which are closely involved in anxiety regulation (27). Furthermore, esketamine has been shown to downregulate the activity of the lateral habenula, a region associated with aversive learning and negative affect (28), which may contribute to its anxiolytic properties. The perioperative period is characterized by heightened sympathetic activation and hypothalamic–pituitary–adrenal axis dysregulation. In this context, esketamine may function not only as a symptomatic anxiolytic but also as a neuromodulatory agent that interrupts the physiological cascade underlying postoperative anxiety.

Despite the overall positive findings regarding emotional symptoms, considerable heterogeneity was present across studies, as indicated by high I^2^ values. This variability likely arises from several sources, including differences in esketamine dosage, route and timing of administration, anesthetic regimens, surgical procedures, and patient characteristics. For example, certain studies employed subanesthetic doses administered during induction, whereas others administered esketamine postoperatively or via continuous infusion. The use of co-administered sedatives, opioids, or anticholinergic agents may have also influenced emotional and cognitive outcomes. Sensitivity analyses using a leave-one-out approach suggested that the pooled estimates were not driven by any single study; however, these analyses are limited and do not fully establish the robustness of the findings. In addition, we conducted clinically relevant subgroup analyses based on surgical type (minimally invasive vs. open surgery) and sex distribution (female-only vs. mixed-sex populations) for SAS and SDS outcomes. These analyses suggested that surgical type may partially contribute to differences in effect estimates, although substantial heterogeneity remained within subgroups. Meta-regression could further help to explore potential sources of heterogeneity, such as dosage, timing, and patient characteristics; however, this was not performed in the present study due to the limited number of included studies and incomplete reporting of key variables, which may reduce the reliability of such analyses.

In contrast to the relatively consistent findings for emotional outcomes, the results regarding cognitive performance were more heterogeneous and less definitive. Pooled analyses using Mini-Mental State Examination and Montreal Cognitive Assessment scores revealed mixed results, with several studies indicating cognitive benefit, while others reported no effect or even transient cognitive impairment. The variability may reflect both methodological and biological factors. Esketamine has been reported to enhance cognitive function acutely by improving attention and psychomotor speed (29). However, it may also impair short-term memory or executive functioning, particularly at higher doses or with repeated exposure (30). These cognitive effects are likely mediated by complex interactions between the prefrontal cortex and subcortical structures and may depend on individual baseline cognitive reserve, neuroinflammatory status, and pharmacodynamic sensitivity.

Notably, one study contributed disproportionately to statistical heterogeneity in both MMSE and MoCA analyses. After exclusion of this outlier, effect estimates shifted in favor of cognitive benefit, with a corresponding reduction in heterogeneity. This finding highlights the importance of methodological quality and the potential impact of individual studies on pooled outcomes. Additionally, the cognitive assessment tools used across studies varied in sensitivity and specificity. The Mini-Mental State Examination, while widely adopted, may be insufficient to detect subtle cognitive changes (31), particularly in domains such as executive function or visuospatial ability. The Montreal Cognitive Assessment, although more comprehensive, was employed in only a limited number of studies (31). Furthermore, the timing of cognitive assessment varied, ranging from early postoperative days to longer-term follow-up, potentially influencing the detection of cognitive decline or recovery. Importantly, a unified definition of postoperative cognitive dysfunction (POCD) was not provided across the included studies. As a result, different trials may have assessed partially different cognitive constructs rather than a strictly consistent POCD endpoint. This lack of standardization further limits comparability across studies.

Several methodological limitations must be acknowledged. The overall quality of the included trials was moderate, with unclear risk of bias in domains such as random sequence generation and allocation concealment in many studies. Blinding procedures were inconsistently reported, and outcome assessors may not have been blinded in all cases, increasing the risk of measurement bias. Furthermore, sample sizes were generally small, limiting statistical power and increasing susceptibility to type II error, particularly for cognitive outcomes. The use of self-reported instruments for mood assessment may also introduce reporting bias, especially in unblinded trials.

Another notable limitation of the present study is the exclusive focus on adult patients (≥18 years old), which precludes the generalization of our findings to the pediatric population undergoing general anesthesia. Perioperative neuropsychiatric complications and cognitive impairment are also prevalent in pediatric surgical patients (32), and the anesthetic management for this population demands a more delicate balance of safety and efficacy. A recent study (33) has reported positive effects of ketamine in pediatric populations, demonstrating that ketamine-based sedation can maintain hemodynamic and respiratory stability, reduce perioperative adverse events, and improve procedural efficiency in pediatric surgical and diagnostic interventions. While this study focused on racemic ketamine rather than esketamine, it provides valuable evidence for the potential application of ketamine derivatives in pediatric perioperative care. Given the structural and pharmacological similarities between esketamine and racemic ketamine, the antidepressant, anxiolytic and neuroprotective properties of esketamine may also be translatable to pediatric patients, which warrants further dedicated investigation.

In terms of clinical implications, the current evidence supports the potential of esketamine as a perioperative intervention to prevent or attenuate affective disturbances. Given the high prevalence of postoperative depression and anxiety and their known associations with poorer recovery trajectories, increased length of hospital staying, and reduced quality of life, the incorporation of esketamine into perioperative care pathways may offer meaningful benefits. Furthermore, perioperative negative emotions can also be alleviated by non-pharmacological approaches such as perioperative counseling, goal-concordant care communication, and caregiver empowerment, which have been recognized as important components of comprehensive perioperative psychological management (34). However, careful patient selection is essential, particularly in elderly individuals or those with cardiovascular instability, as esketamine is associated with transient increases in blood pressure and heart rate. Importantly, safety outcomes such as postoperative nausea and vomiting, cardiovascular events, and postoperative delirium were not systematically assessed across included studies and therefore could not be quantitatively synthesized, which should be considered when interpreting the present findings. Additional studies are needed to determine optimal dosing strategies, treatment duration, and patient subgroups most likely to benefit.

Further well-designed randomized controlled trials are warranted to validate these findings and improve the overall certainty of evidence. Studies should incorporate long-term follow-up to assess the durability of esketamine’s neuropsychiatric effects and consider stratification by preoperative mental health status, cognitive baseline, and inflammatory markers. The integration of neuroimaging, electrophysiology, and biomarker analyses may help elucidate the mechanisms underlying individual response variability. Moreover, head-to-head comparisons with conventional antidepressants or anxiolytics would help define esketamine’s unique role in perioperative neuropsychiatric management.

## 5 Conclusion

In conclusion, this meta-analysis suggests that esketamine may be associated with reductions in postoperative depressive and anxiety symptoms in patients undergoing general anesthesia, while its cognitive benefits remain unclear owing to high heterogeneity and very low evidence certainty. Therefore, while esketamine shows potential in perioperative neuropsychiatric management, further well-designed, large-scale studies are needed to confirm its efficacy and safety.

## Data Availability

The data used to support the findings of this study are available in the article.
