# Supplementary material for: Esketamine for negative emotions and cognitive function in general anesthesia: a systematic review and meta-analysis
Source: Front Med (Lausanne). 2026 Jun 12;13:1816437. doi: 10.3389/fmed.2026.1816437 (PMC13305730; doi:10.3389/fmed.2026.1816437)
Supplement: Supplementary file 1 [file Table_1.docx]

**Appendix Table 1**

| Database | Retrieval strategy |
| --- | --- |
| Pubmed | ("Esketamine"[Mesh] OR "Esketamine" OR "S-Ketamine" OR "S Ketamine") AND ("Anesthesia, General"[Mesh] OR "general anesthesia" OR "under general anesthesia" OR "postoperative patients") AND (("Emotions"[Mesh] OR "emotion" OR "mood" OR "negative emotions" OR "anxiety"[Mesh] OR "depression"[Mesh] OR "anxiety" OR "depression") OR ("Cognition"[Mesh] OR "Cognitive Dysfunction"[Mesh] OR "cognitive function" OR "cognitive impairment" OR "postoperative cognitive dysfunction" OR "POCD")) |
| Embase | ('esketamine'/exp OR esketamine OR 's-ketamine') AND ('general anesthesia'/exp OR 'postoperative patient'/exp OR 'general anesthesia' OR 'postoperative patients') AND (('negative emotion' OR 'emotional disorder'/exp OR 'anxiety'/exp OR 'depression'/exp OR 'mood disorder'/exp) OR ('cognition'/exp OR 'cognitive impairment'/exp OR 'postoperative cognitive dysfunction'/exp OR 'POCD' OR 'cognitive function')) |
| Web of Science | TS=("Esketamine" OR "S-Ketamine" OR "S Ketamine") AND TS=("general anesthesia" OR "postoperative patients" OR "after general anesthesia") AND TS=("negative emotion" OR "emotional state" OR "anxiety" OR "depression" OR "cognitive function" OR "cognitive impairment" OR "postoperative cognitive dysfunction" OR "POCD") |
| Cochrane library | ("Esketamine" OR "S-Ketamine" OR "S Ketamine") AND ("general anesthesia" OR "postoperative patients") AND ("negative emotion" OR "anxiety" OR "depression" OR "cognitive function" OR "cognitive impairment" OR "POCD") |
| CNKI | SU = ('esketamine' + 'S-ketamine') * ('general anesthesia' + 'general anaesthesia' + 'postoperative') * ('negative emotions' + 'anxiety' + 'depression' + 'cognitive function' + 'postoperative cognitive dysfunction' + 'POCD') |
